# Metabolic recycling of storage lipids promotes squalene biosynthesis in yeast

**DOI:** 10.1186/s13068-022-02208-9

**Published:** 2022-10-12

**Authors:** So-Hee Son, Jae-Eung Kim, Soo Young Moon, In-Seung Jang, Byung Jo Yu, Ju Young Lee

**Affiliations:** 1 Research Center for Bio-Based Chemistry, Korea Research Institute of Chemical Technology (KRICT), Ulsan, 44429 Republic of Korea; 2 School of Interdisciplinary Bioscience and Bioengineering, Pohang University of Science and Technology (POSTECH), Pohang, Gyeongbuk 37673 Republic of Korea; 3 Intelligent Sustainable Materials R&D Group, Research Institute of Sustainable Manufacturing System, Korea Institute of Industrial Technology (KITECH), Cheonan, 31056 Republic of Korea

**Keywords:** Lipid droplet, Metabolic recycling, Yeast, Terpene, Squalene, Metabolic engineering, Synthetic biology

## Abstract

**Background:**

Metabolic rewiring in microbes is an economical and sustainable strategy for synthesizing valuable natural terpenes. Terpenes are the largest class of nature-derived specialized metabolites, and many have valuable pharmaceutical or biological activity. Squalene, a medicinal terpene, is used as a vaccine adjuvant to improve the efficacy of vaccines, including pandemic coronavirus disease 2019 (COVID-19) vaccines, and plays diverse biological roles as an antioxidant and anticancer agent. However, metabolic rewiring interferes with inherent metabolic pathways, often in a way that impairs the cellular growth and fitness of the microbial host. In particular, as the key starting molecule for producing various compounds including squalene, acetyl-CoA is involved in numerous biological processes with tight regulation to maintain metabolic homeostasis, which limits redirection of metabolic fluxes toward desired products.

**Results:**

In this study, focusing on the recycling of surplus metabolic energy stored in lipid droplets, we show that the metabolic recycling of the surplus energy to acetyl-CoA can increase squalene production in yeast, concomitant with minimizing the metabolic interferences in inherent pathways. Moreover, by integrating multiple copies of the rate-limiting enzyme and implementing N-degron-dependent protein degradation to downregulate the competing pathway, we systematically rewired the metabolic flux toward squalene, enabling remarkable squalene production (1024.88 mg/L in a shake flask). Ultimately, further optimization of the fed-batch fermentation process enabled remarkable squalene production of 6.53 g/L.

**Conclusions:**

Our demonstration of squalene production via engineered yeast suggests that plant- or animal-based supplies of medicinal squalene can potentially be complemented or replaced by industrial fermentation. This approach will also provide a universal strategy for the more stable and sustainable production of high-value terpenes.

**Supplementary Information:**

The online version contains supplementary material available at 10.1186/s13068-022-02208-9.

## Background

Metabolic rewiring in microbes has been proposed as a promising strategy for the economical and sustainable production of valuable medicinal compounds that are found in trace quantities in nature [1–4]. Metabolic pathways have been rewired to unlock metabolic bottlenecks by enhancing the supply of metabolic building blocks or reducing metabolic flux-competing reactions toward the desired compounds [5]. However, metabolic rewiring interferes with inherent metabolic pathways, often in a way that impairs the cellular growth and fitness of the microbial host. In particular, as the key starting molecule for producing various compounds, acetyl-CoA is involved in numerous biological processes with tight regulation to maintain metabolic homeostasis, which limits redirection of metabolic fluxes toward desired products [1, 6].

**Fig. 1:**
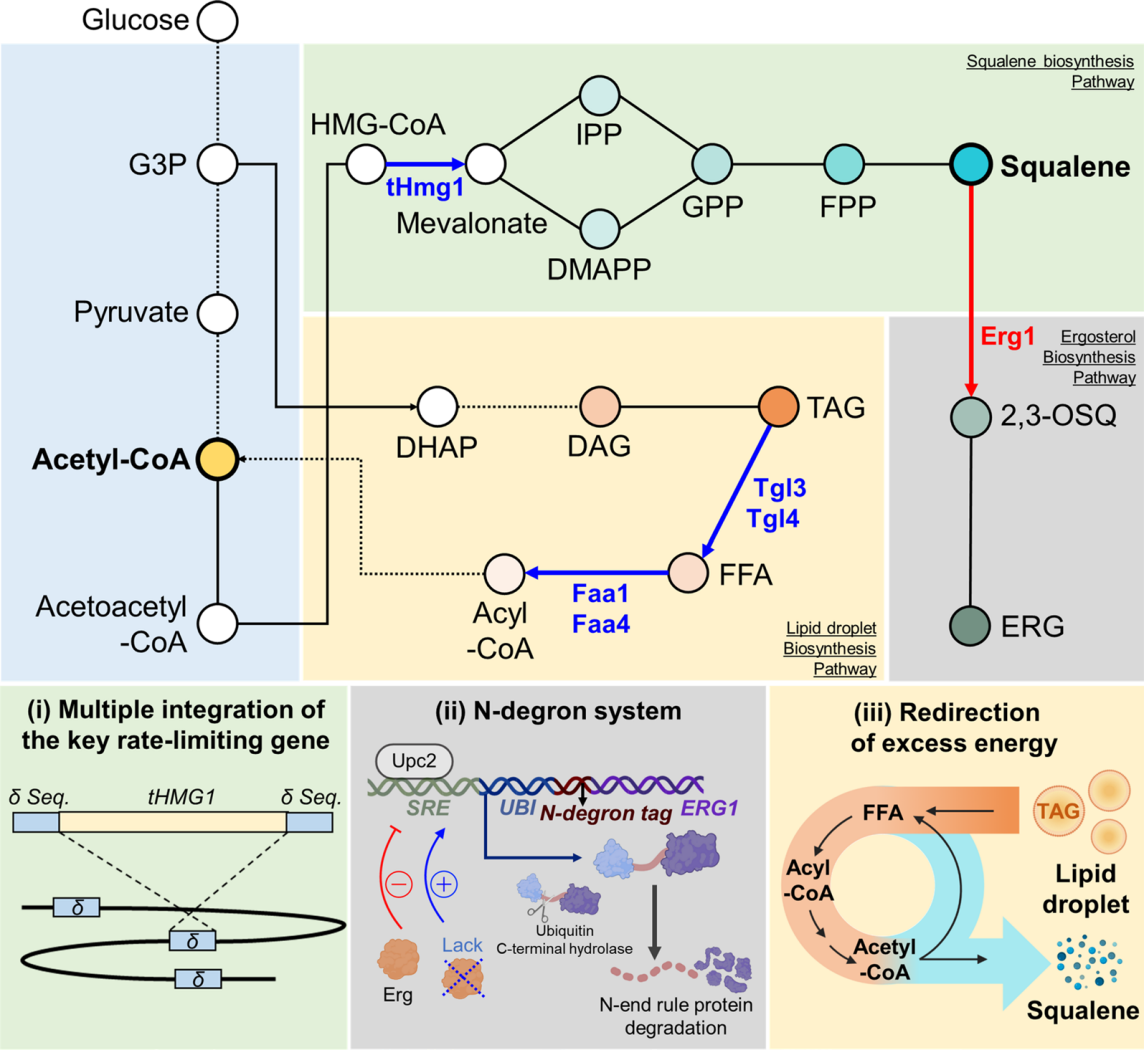
Metabolic rewiring for high-level production of squalene in yeast. Schematic illustration of metabolic engineering strategies for squalene production from glucose, which include (i) optimizing the key rate-limiting enzyme expression by multiple integration (green box), (ii) controlling the competitive ergosterol biosynthesis pathway using the N-degron-dependent protein degradation system (gray box), and (iii) enhancing the availability of the precursor acetyl-CoA via the metabolic recycling of the surplus energy of LDs (yellow box). Specified pathways related to squalene biosynthesis are highlighted in different colored boxes (top), and corresponding metabolic engineering strategies are shown in boxes with the same color code (bottom). Solid lines or arrows indicate a single metabolic reaction, and dashed lines or arrows indicate multiple reactions. Increased and decreased proteins are shown in blue and red, respectively. *G3P* glycerol-3-phosphate, *HMG-CoA* 3-hydroxy-3-methylglutaryl-CoA, *IPP* isopentenyl pyrophosphate, *DMAPP* dimethylallyl pyrophosphate, *GPP* geranyl diphosphate, *FPP* farnesyl pyrophosphate, *2,3-OSQ* 2,3-oxidosqualene, *ERG* ergosterol, *DHAP* dihydroxyacetone phosphate, *DAG* diacylglycerol, *TAG* triacylglycerol, *FFA* free fatty acid, *tHmg1* truncated 3-hydroxy-3-methylglutaryl-CoA reductase 1, *Erg1* squalene epoxidase, *Tgl3/4* triacylglycerol lipase, *Faa1/4* acyl-CoA synthetase, *Upc2* sterol regulatory element-binding protein, *SRE* sterol regulatory element, *UBI* ubiquitin

Lipid droplets (LDs) are energy storehouses of surplus metabolic energy and lipids, supplying the cells with metabolic building blocks such as acetyl-CoA for energy generation when the cells are starved [7, 8]. This implies that the breakdown of surplus lipids in LDs can provide sufficient acetyl-CoA for cellular growth and overall fitness of the host cells, allowing the concomitant overproduction of the desired target compounds. We thus explored the metabolic recycling of acetyl-CoA from the surplus energy in LDs for the production of medicinal terpenes while minimizing the metabolic interferences in the inherent pathways. Here, we performed systematic metabolic rewiring of *Saccharomyces cerevisiae* toward squalene production (Fig. 1). As a model medicinal terpenes, squalene is a high-value natural compound that has been shown to play diverse biological roles as an antioxidant and anticancer agent and has been employed as a vaccine adjuvant to improve the efficacy of vaccines, including pandemic coronavirus disease 2019 (COVID-19) vaccines [9, 10]. Although increased consumer demand has prompted the development of microbial bioprocesses for squalene production, these cannot meet the growing demand for squalene.

In this study, we first controlled the intrinsic mevalonate (MVA) pathway by multiple integration of the rate-limiting enzyme tHmg1 (a cytosolic non-feedback-inhibited 3-hydroxy-3-methylglutaryl-CoA reductase) into the genome of our platform yeast strain for terpene production (SQ00) [11]. Subsequently, driven by an N-degron-dependent protein degradation strategy [12, 13], we downregulated Erg1 (a squalene epoxidase catalyzing the oxidation of squalene to 2,3-oxidosqualene) activity. We further rewired LD metabolism to improve the acetyl-CoA supply, which resulted in significantly increased production of squalene (1024.88 mg/L by batch fermentation in a shake flask). Ultimately, further optimization of the fed-batch fermentation process enabled remarkable squalene production of 6.53 g/L. Our study offers an efficient strategy to enhance squalene production and might also be applicable for the production of other valuable compounds.

## Results and discussion

### Construction of a squalene-overproducing pathway in *S. cerevisiae*

Squalene is naturally synthesized through the MVA pathway, involving multiple enzymes, in yeast (Fig. 1). Metabolic fluxes through the MVA pathway are under tight feedback regulation from pathway intermediates or downstream products [14, 15]; for instance, HMG, free CoA, and NAD(P)^+^/NADPH inhibiting HMG–CoA reductase (Hmg1) [16] and sterol inhibiting squalene epoxidase (Erg1) [13] catalyze the rate-limiting step, thereby controlling the squalene biosynthesis flux. Therefore, to construct a strain for high-level production of squalene, we investigated the effects of the rate-limiting enzymes in squalene biosynthesis.

**Fig. 2:**
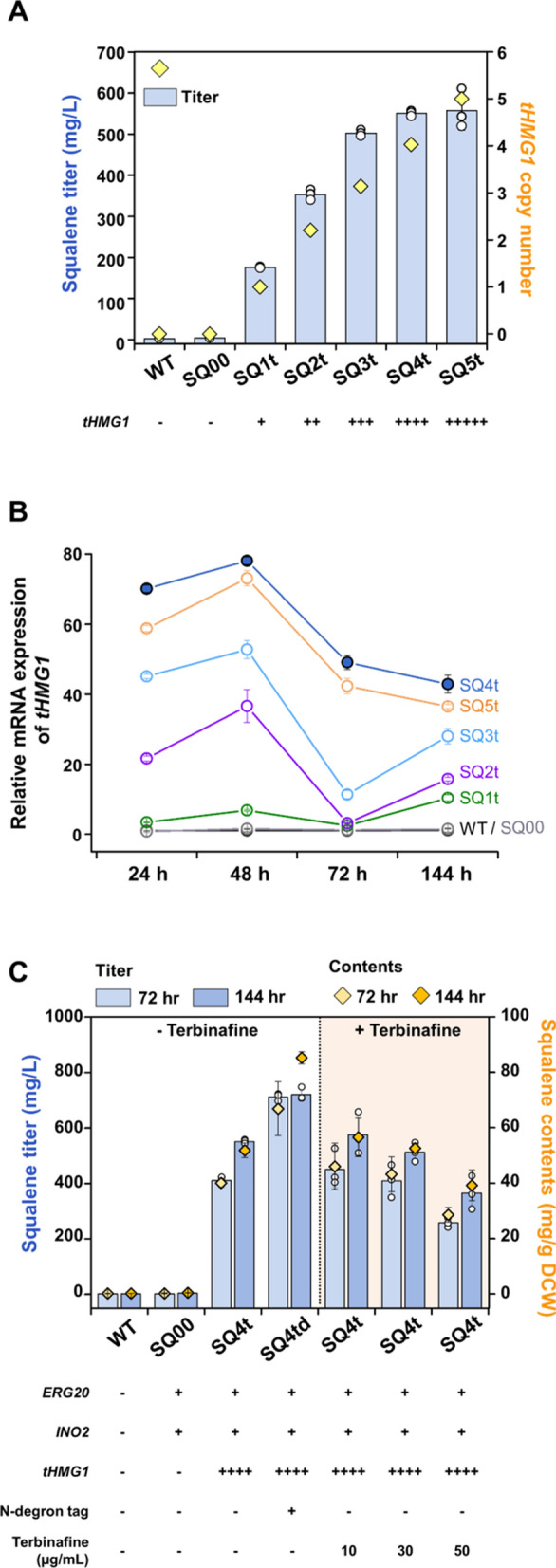
Squalene production in engineered yeast strains. **A** Construction of squalene-producing strains by multicopy integration of the *tHMG1* gene into the SQ00 strain, our platform strain for terpene production. Increasing the *tHMG1* copy number (up to 4 copies) comparably increased squalene production. **B** Changes in the relative mRNA expression level of the *tHMG1* gene. The relative expression levels of the *tHMG1* gene from the engineered strains were based on the expression level of the *tHMG1* gene from the WT strain at 24 h. The copy numbers of the integrated *tHMG1* gene is positively correlated with its expression up to 4 copies. **C** Partial inhibition of Erg1 activity reduced metabolic flux toward ergosterol synthesis to enhance squalene production. When Erg1 was tagged with an N-degron in the SQ4t stain (resulting in the SQ4td strain), degradation of Erg1 successfully redirected metabolic flux toward squalene production. In contrast, although partial inhibition of Erg1 activity with the addition of its inhibitor terbinafine was likely effective for squalene production, a pronounced reduction in squalene production with increasing terbinafine concentration was observed. Yeast cells were grown in shake flasks with YSC minimal medium with 2% (w/v) glucose at 30 °C. All data are presented as the mean ± standard deviation of biological triplicates. WT, wild type. Each plus (+) symbol indicates one copy of the gene integrated into the genome

First, we overexpressed a truncated *HMG1* (*tHMG1*) gene encoding the catalytic domain of Hmg1, which has been reported as the major rate-limiting enzyme to enhance metabolic fluxes in the MVA pathway and squalene production [17], in a yeast biosynthetic platform for terpene production developed by our group, the SQ00 strain [11] (Additional file 1: Table S1). The strain SQ00, designed to increase terpene production, incorporated (i) overexpression of FPP synthetase (Erg20), which supplies sufficient FPP for terpene synthesis [11, 18], and (ii) expansion of the endoplasmic reticulum (ER) by overexpressing a key ER size regulatory factor (Ino2) for functional assembly of the terpene synthesis pathway [11]. We integrated the *tHMG1* gene into a multicopy δ-sequence of the SQ00 genome (Fig. 1), generating a series of squalene-producing strains in which the yields of squalene production varied from 11.10 mg/L to 550.89 mg/L after 144 h of fermentation (Fig. 2A; Additional file 1: Fig. S1). The δ-integration strategy has been widely used for the overexpression of heterologous genes to construct biosynthesis pathways, as it can simultaneously achieve random multicopy integration of target genes [19, 20].

The copy numbers of the integrated *tHMG1* gene were investigated using quantitative PCR analysis (Fig. 2A). The transformants with squalene production titers over 500 mg/L contained 4 copies of the *tHMG1* gene. Furthermore, we found that as the copy numbers of the *tHMG1* gene increased, its transcription level gradually increased up to 4 copies, but rather decreased when the copy numbers are further increased (Fig. 2B). This observation indicates that the copy numbers of the integrated *tHMG1* gene is indeed positively correlated with its expression as well as squalene production titer up to a certain copy number level. Thus, among those strains, one strain with the highest squalene titer (550.89 mg/L) and the *tHMG1* expression level (4 copies) as well was selected and referred to as the SQ4t strain, which had an almost 133-fold greater squalene titer than the control strain SQ00 (4.14 mg/L). The variations in squalene production among strains with the same copy number of *tHMG1* were speculated to be affected by different δ-integration sites, because it was recently shown that the position effect accounts for increased variability in gene expression levels [21].

Second, to further improve squalene production by the SQ4t strain, we modulated the ergosterol biosynthesis pathway, which converts squalene to ergosterol (Fig. 1). Ergosterol is an essential membrane component, with tightly controlled homeostasis in yeast. Accordingly, as most genes required for ergosterol synthesis are vital for cell growth, they cannot be deleted. Transcriptional downregulation of *ERG1*, the first enzyme catalyzing the epoxidation of squalene to 2,3-oxidosqualene in the ergosterol biosynthesis pathway, also leads to growth defects, causing failure to engineer yeast suitable for industrial-scale production of squalene [12, 13]. Although partial inhibition of Erg1 activity with the addition of its inhibitor terbinafine was effective for squalene accumulation, as reported previously [22], it showed a pronounced reduction in cell growth and squalene titer with increasing terbinafine concentration (Fig. 2C; Additional file 1: Table S3). In addition, terbinafine is a highly lipophilic supplement that is difficult to remove through the downstream process of purification for squalene production.

Therefore, we utilized the N-degron-mediated protein degradation strategy to reduce Erg1 activity concomitant with the maintenance of ergosterol flux for normal cell growth and coupled it with an ergosterol-responsive transcription modulator circuit (Fig. 1) [23]. The N-degron tag is a ubiquitin moiety and a destabilizing sequence enriched with lysine and asparagine, which are sequentially fused to the N-terminus of a target protein [23, 24]. The resulting fusion protein is hydrolyzed by ubiquitin C-terminal hydrolase, and the destabilizing sequence is exposed following N-rule protein degradation. Therefore, the N-degron tag can result in a reduced half-life or deletion-like phenotype of the target protein by inducing proteasomal proteolysis. In this study, to reduce metabolic flux toward ergosterol synthesis, we modified the genomic *ERG1* gene by fusing the N-degron to the Erg1p N-terminus (Additional file 1: Table S2). Furthermore, as noted, the *ERG1* gene under the control of an ergosterol-responsive promoter is regulated inversely proportionally to ergosterol concentration [25]. Consequently, degron-tagged *ERG1* expression was paired with the ergosterol-responsive promoter, thus increasing squalene accumulation while ensuring sufficient metabolic flux to essential downstream ergosterol synthesis.

The resulting strain expressing the N-degron-tagged Erg1 in the SQ4t strain (SQ4td strain) produced 720.47 mg/L squalene after 144 h of fermentation, and this value exhibited an ~ 31% increase over that produced by the control SQ4t strain (550.89 mg/L), without a reduction in cell growth (Fig. 2C; Additional file 1: Table S3). This result indicates that with the aim of minimizing carbon flux toward ergosterol synthesis, the N-degron-mediated degradation of Erg1 successfully reduces carbon flux toward ergosterol synthesis and redirects the carbon flux into squalene production, similar to the effect of using terbinafine but without compromising cell growth.

### Redirection of LD metabolism to boost squalene production

Neutral lipids stored in LDs are mobilized in the form of free fatty acids (FFAs) for energy production during nutrient deprivation or for phospholipid synthesis during the high demand of membrane production [26, 27]. FFAs released from LDs through lipolysis can be redirected toward the supply of acetyl-CoA, a key metabolic building block for many biochemical reactions. Previous studies reported that hydrolyzing TAG to fatty acids (FAs) and diacylglycerol (DAG) may influence acetyl-CoA availability via subsequent metabolism through β-oxidation (Fig. 1) [28–30]. For this reason, we hypothesized that recycling of FAs stored in LDs can support an increase in the acetyl-CoA supply, favoring squalene production.

**Fig. 3:**
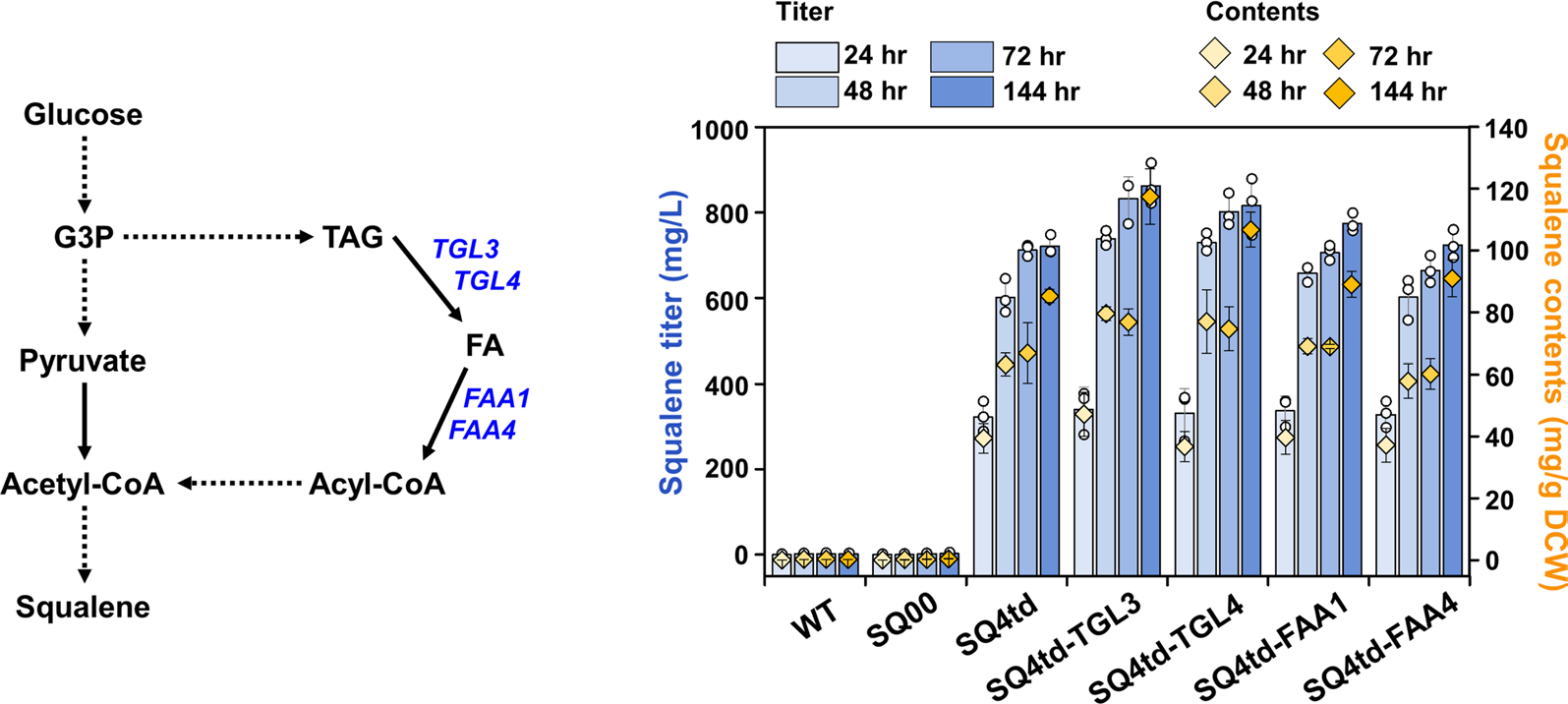
Improved squalene production through redirection of LD metabolism. To ensure sufficient acetyl-CoA precursor supply for squalene production, neutral lipids stored in LDs were degraded and redirected toward the supply of acetyl-CoA in the SQ4td strain. The *TGL3* or *TGL4* gene involved in FA supply was overexpressed, and the gene encoding acyl-CoA synthetase, *FAA1* or *FAA4*, was overexpressed to produce acyl-CoA from FAs. Yeast cells were cultivated in shake flasks with YSC medium containing 2% (w/v) glucose at 30 °C for 144 h. All data are presented as the mean ± standard deviation of biological triplicates

To verify this hypothesis, we overexpressed four different genes involved in TAG lipolysis or acyl-CoA synthesis in the SQ4td strain. In detail, we overexpressed the *TGL3* or *TGL4* gene encoding the main TAG-lipase involved in FA supply [31], generating SQ4td-TGL3 and SQ4td-TGL4. The *FAA1* or *FAA4* gene (encoding a acyl-CoA synthetase), which produces acyl-CoA from FAs [32], was overexpressed in SQ4td to generate SQ4td-FAA1 or SQ4td-FAA4. The level of squalene production was the highest (862.62 mg/L) in the SQ4td-TGL3 strain after 144 h of cultivation, which was approximately 20% higher than that by SQ4td (720.47 mg/L) (Fig. 3). In addition, the SQ4td-TGL4 and SQ4td-FAA1 strains produced higher levels of squalene (816.48 and 774.07 mg/L, respectively) than SQ4td. However, squalene production in the SQ4td-FAA4 strain was not increased (723.90 mg/L), which is consistent with a previous report demonstrating that Faa1 rather than Faa4 acts as the predominant fatty acyl-CoA synthetase [32]. In addition, the growth of LD metabolism-engineered strains was similar to that of SQ4td, suggesting that the overexpression of the four genes for recycling LD-dependent FAs did not inhibit cell growth (Additional file 1: Table S4). In an attempt to further increase squalene production, we combined overexpression of the *TGL3* and *TGL4* genes (SQ4td-TGL3/4 strain), but no obvious improvement in squalene production was observed (832.63 mg/L) (Additional file 1: Table S4).

**Fig. 4:**
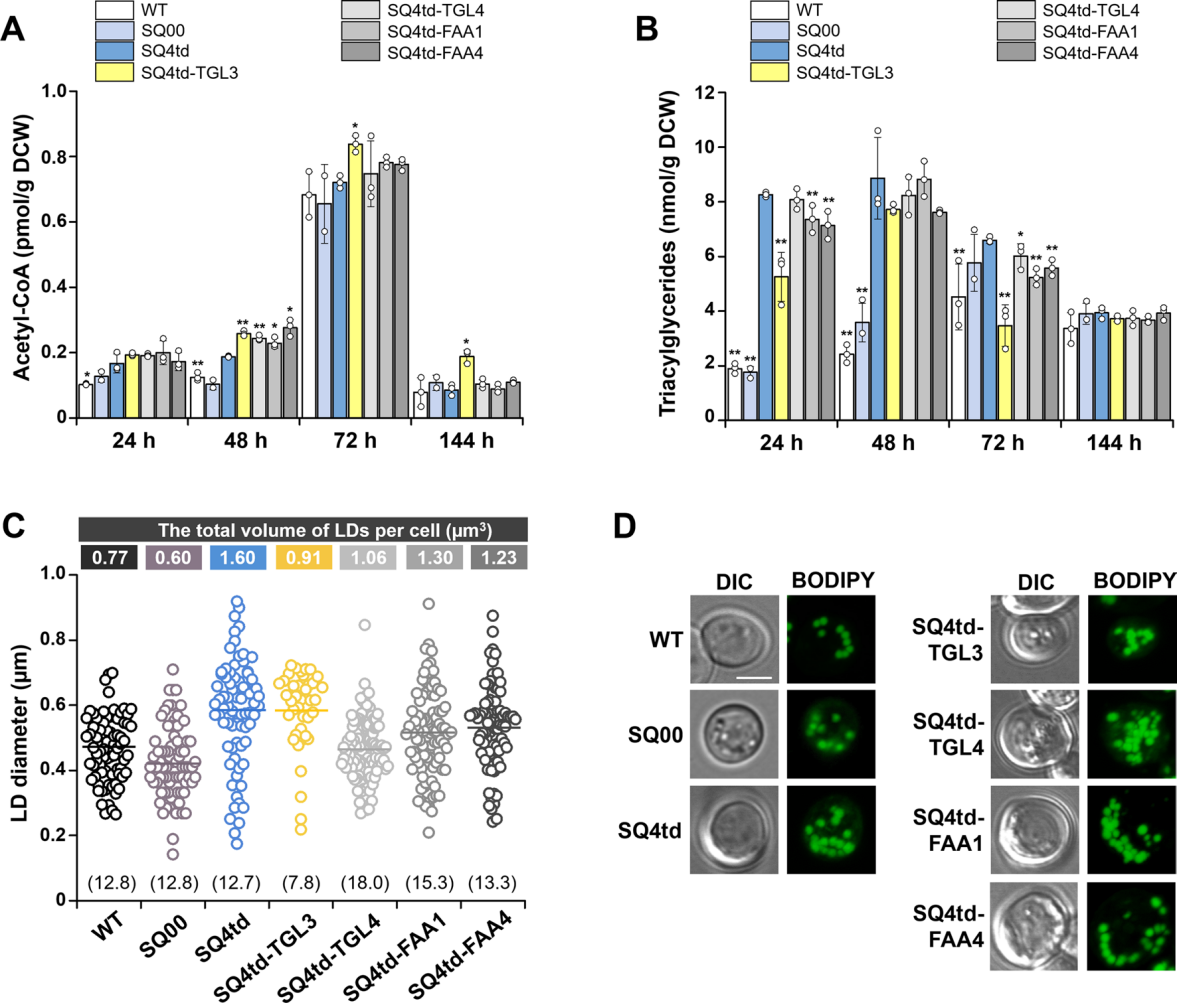
Supply of acetyl-CoA driven by degradation of TAG in LD metabolism-engineered strains.** A** Acetyl-CoA and **B** TAG levels of the LD metabolism-engineered strains. The acetyl-CoA level of the LD metabolism-engineered strains slightly increased with a concomitant decrease in TAG levels compared to those of the control SQ4td strain. **C** Quantification of the size and number of LDs in the LD metabolism-engineered strains. The graph depicts scatter plots of morphological changes in the LDs of the LD metabolism-engineered strains (n = 6). For the scatter plots, medians with interquartile ranges are displayed. The numbers in parentheses indicate the number of LDs in each engineered strain. **D** Differential interference contrast and confocal fluorescence microscopy images of the LDs in the engineered yeast strains. All strains were cultured in shake flasks with YSC medium containing 2% (w/v) glucose for 144 h at 30 °C. All the data represent the mean of biological triplicates, and error bars indicate the standard deviation. An asterisk indicates that the value is significantly different (*P < 0.05 and **P < 0.01) from that of the respective control SQ4td strain. Scale bar: 2.5 μm

Thus, we performed additional analyses for only single mutants except for the SQ4td-TGL3/4 strain. First, we evaluated whether acetyl-CoA content was increased by rewiring LD metabolism. The level of acetyl-CoA in SQ4td-TGL3 was highly increased by ~ 24% (0.84 pmol/g dry cell weight (DCW)) at 72 h compared with that in the SQ4td strain (0.68 pmol/g DCW) (Fig. 4A). Moreover, we observed that the highest level of acetyl-CoA was sustained in the SQ4td-TGL3 strain during cultivation for 144 h, which was consistent with the finding that the SQ4td-TGL3 strain produced squalene most productively. This result might be due to an increase in acetyl-CoA supply by TAG mobilization or degradation via a cascade of hydrolysis reactions from TAG and DAG to FAs.

Next, to further clarify the effect of acetyl-CoA supply on TAG degradation, TAG levels were quantified in the engineered strains. The level of TAG was decreased by ~ 47% (3.47 nmol/g DCW) in the highest squalene-overproducing strain SQ4td-TGL3 compared with that in the SQ4td strain (6.59 nmol/g DCW) at 72 h (Fig. 4B). The effect of TAG lipolysis was maintained from 24 to 72 h of cultivation in the SQ4td-TGL3 strain. Consistent with the decreased TAG level, the SQ4td-TGL3 strain had significantly fewer LDs than the SQ4td strain; the number of LDs in the SQ4td-TGL3 strain decreased by ~ 39% (7.8 LDs per cell), with no significant difference in the average diameter of the LDs (D_avg_ ~ 0.58 µm), resulting in an ~ 43% decrease in the total LD volume per cell (0.91 µm^3^) compared to those in the SQ4td strain (12.7 LDs per cell, average diameter of 0.58 µm and volume per cell of 1.60 µm^3^) (Fig. 4C, D; Additional file 1: Fig. S2). Consequently, LD metabolism rewiring by *TGL3* overexpression increased the acetyl-CoA supply, which could explain its larger contribution to squalene overproduction.

### Optimization of culture conditions for enhanced squalene production

For the engineered yeast strain with the most promising performance for squalene production, the choice of carbon source was important for the production efficiency. Ethanol is often used as the main carbon source in fed-batch fermentation due to increased titers in the production of many terpenes [33]. Glycerol is another potential source for yeast due to its higher reduced state of carbon compared to that of other sugars [34]. However, glucose is still a widely used carbon source in industrial terpene production.

**Fig. 5:**
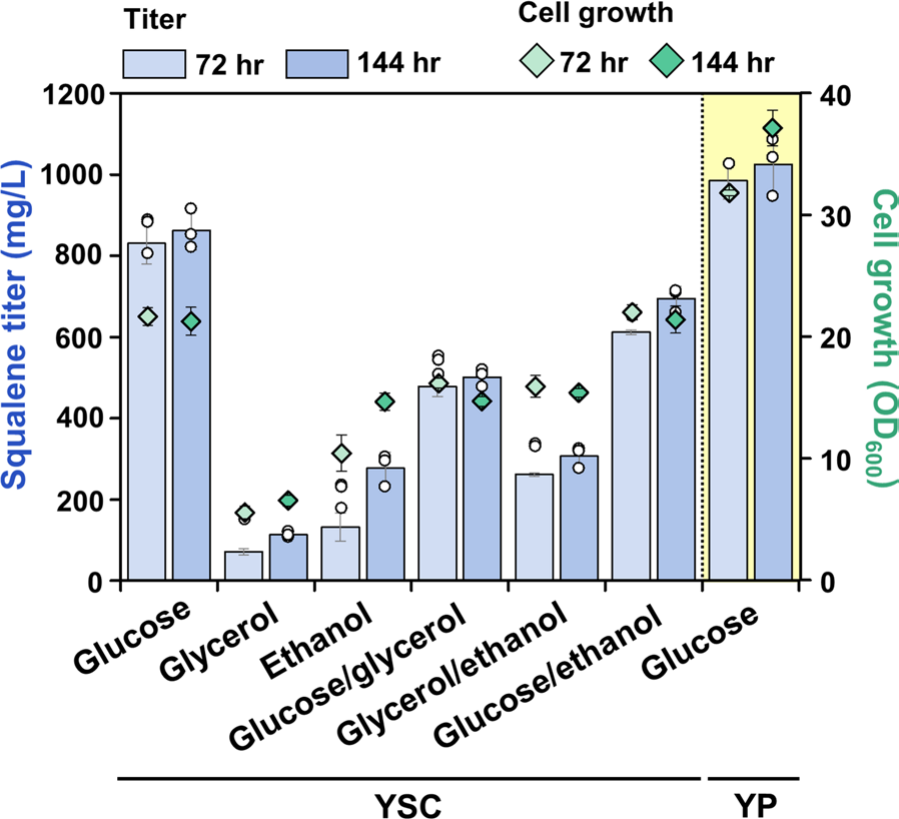
Effect of the carbon source and cultivation medium on squalene production in the squalene-overproducing strain SQ4td-TGL3. Analysis of the SQ4td-TGL3 strain for the production of squalene using three different carbon sources in YSC minimal or YP complex medium. The highest squalene titer was achieved in YP complex medium containing 2% (w/v) glucose at 144 h of cultivation. The strain was cultivated in shake flasks with YSC or YP medium containing 2% (w/v) glucose, glycerol, or ethanol as the sole carbon source or a mixture of the two carbon sources (1:1, 1% v/v each) for 144 h at 30 °C. All data represent the mean ± standard deviation of biological triplicates

Therefore, we tested three different carbon sources to determine which would be optimal for the production of squalene. The best strain SQ4td-TGL3 was cultivated in shake flasks with glucose, glycerol, or ethanol (2%) as the carbon source, and the highest titer of 862.62 mg/L squalene was produced from glucose as a sole carbon source after 144 h of cultivation (Fig. 5). In contrast, we observed significantly lower titers of squalene (115.90 and 279.75 mg/L after 144 h of cultivation) from glycerol and ethanol, respectively.

Previously, studies have shown that when mixing carbon sources, cells tend to coutilize the carbon sources, and the growth rate or the yield of target products is higher than that with each individual source [35]. Thus, we further investigated squalene production using mixed carbon sources, namely, glucose/glycerol, glycerol/ethanol, and glucose/ethanol mixed carbon sources (1:1, 1% v/v each) (Fig. 5). However, when cultivated on a mixture of two carbon sources, the strain SQ4td-TGL3 produced lower levels of squalene: 505.37 mg/L on glucose/glycerol, 308.53 mg/L glycerol/ethanol, and 695.61 mg/L glucose/ethanol. This result indicates that glycerol and ethanol were not suitable as the carbon source in our strain and that utilization of these nonfermentable carbon sources might be inhibited in the presence of glucose [36, 37].

**Fig. 6:**
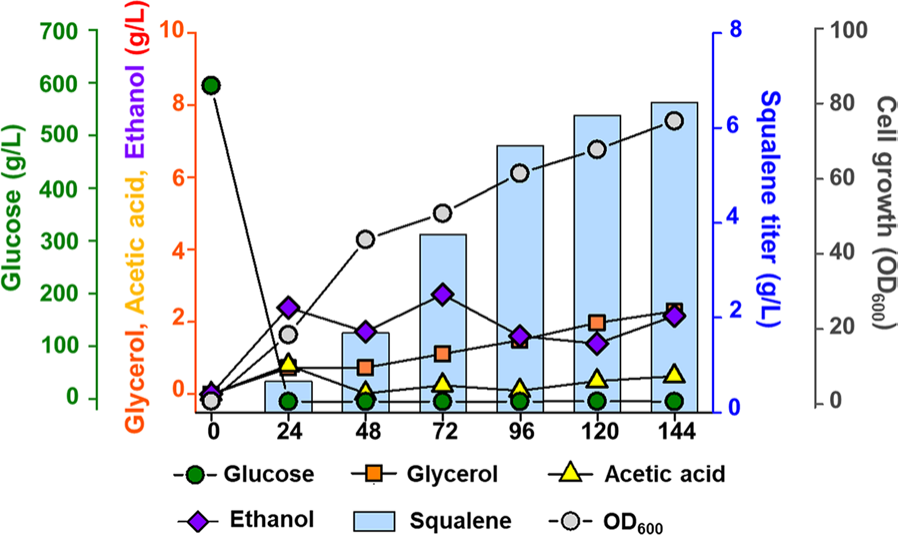
Fed-batch fermentation of the squalene-overproducing SQ4td-TGL3 strain. Time courses of metabolite titers and cell growth during fed-batch fermentation are shown. Fermentation was performed in a 5 L bioreactor containing 2 L of YP medium at 30 °C with an airflow rate of 1 v.v.m. Symbols in fermentation profiles denote squalene (blue bars), glucose (green circles), glycerol (orange squares), acetic acid (yellow triangles), and ethanol concentrations (purple diamonds) and cell growth (gray circles)

In general, yeast cells grow more vigorously in complex media than in defined media; since complex media include unknown ingredients, they are rich in nutrients and other necessary elements to support cellular growth [38, 39]. Notably, when cultivated in the complex medium with glucose, the strain SQ4td-TGL3 showed a 26% higher squalene titer (1024.88 mg/L) after 144 h of cultivation compared to that in the defined medium (Fig. 5). Finally, in a 5-L bioreactor, we realized greater improvements in the final production titer, which reached 6.53 g/L squalene in the fed-batch mode using the complex medium with glucose as the carbon source (Fig. 6).

## Conclusions

Squalene is a crucial intermediate and precursor for valuable bioactive compounds used in the food, drug, chemical and cosmetic industries. Given the growing demand for squalene, three to six million deep sea sharks are slaughtered each year. A global health crisis, such as the ongoing COVID-19 pandemic, has resulted in the further increased need for squalene, which is an ingredient currently used in influenza vaccines. To overcome this unsustainable practice, we engineered baker’s yeast to produce the high-value chemical squalene, starting from simple sugars. This was accomplished by (i) overexpressing the native rate-limiting enzyme encoded by the *tHMG1* gene, (ii) controlling the competitive ergosterol biosynthesis pathway using the N-degron-dependent protein degradation strategy, (iii) improving acetyl-CoA supply via the metabolic recycling of the surplus energy of LDs, and (iv) optimizing the fermentation medium, including the carbon source and the cultivation medium. Overall, we systematically rewired the metabolic flux toward squalene, enabling remarkable squalene production (1024.88 mg/L in the shake flask and 6.53 g/L in the fed-batch fermenter). Our demonstration of squalene production via engineered yeast suggests that plant- or animal-based supplies of medicinal squalene can potentially be complemented or replaced by industrial fermentation.

## Methods

### Plasmid and strain construction

All plasmids, strains and primers are listed in Additional file 1: Tables S1 and S2. The plasmids used in this study were generated via insertion of the gene fragment, which was amplified from the yeast genomic DNA with corresponding primer pairs and digested with restriction enzymes, into pUC57-URA3-derived vectors for strain construction. Gene modifications were introduced by the URA3-blaster genetic disruption method [40]. Recombination cassettes for gene integration or promoter replacement were amplified by PCR from pUC57-URA3-derived vectors containing a gene of interest or the appropriate promoter, respectively, with primer pairs introducing regions homologous to the target recombination site. The yeast plasmids were transformed into the recombinant strains by the standard LiAc/ssDNA/PEG method [41].

### Shake flask fermentation for squalene production

A total of 50 mL of yeast synthetic complete (YSC) medium (0.19% yeast synthetic dropout medium without uracil and 0.67% yeast nitrogen base without amino acids) supplemented with 2% (w/v) glucose was used to culture engineered yeast strains for the production of squalene. First, engineered strains were grown on YSC agar plates with 2% (w/v) glucose lacking uracil. A yeast colony was inoculated into 50 mL conical tubes with 10 mL of YSC seed medium. After overnight cultivation at 30 °C with shaking at 250 rpm, the seed cultures were inoculated into 250 mL flasks with 50 mL of YSC medium containing a 2% carbon source to reach an initial optical density (OD_600_) of 0.5 and then cultivated for 6 days at 30 °C with shaking at 250 rpm. For the carbon sources in each medium, the following concentrations were applied: 2% (w/v) glucose, 2% (v/v) glycerol, and 2% (v/v) ethanol. All flask fermentations were performed in three independent experiments.

### Determination of gene copy number and gene expression level

To determine the copy numbers of the *tHMG1* gene, quantitative PCR (qPCR) was performed with *tHMG1*-specific primers and iTaq Universal SYBR Green Supermix (Bio-Rad, CA, USA). qPCR was performed on a CFX96™ Real-Time PCR Detection System (Bio-Rad, CA, USA) with the following conditions: initial denaturation at 95 °C for 30 s, followed by 40 cycles of 95 °C for 5 s and 60 °C for 15 s [42, 43]. The *TAF10* gene was used as a reference control. The crossing point (Cp) values were determined using Bio-Rad CFX Manager software (Bio-Rad, CA, USA).

For analyzing gene expression level of the *tHMG1* gene, RNA was extracted from the cells using RNeasy kit (Qiagen, Hilden, Germany) at the time intervals as indicated. Then, the cDNA was synthesized with a Superscript III First-Strand Synthesis System (Life Technologies, OR, USA) according to the manufacturer’s instructions. The relative gene expression level was quantified by the comparative threshold cycle (2^−∆∆Ct^) method [44]. The primers used are listed in Additional file 1: Table S1. All experiments were performed in triplicate.

### Fed-batch fermentation for squalene production

Fed-batch fermentations were performed in a 5 L fermenter (CNS, Daejeon, Korea). The initial working volume was 2 L of YP medium (10 g/L yeast extract and 20 g/L peptone) supplemented with 2% (w/v) glucose and 200 mL of overnight preculture in the same medium. Fed-batch fermentation was carried out at 30 °C with an agitation speed of 600 rpm and an airflow rate of 1 v.v.m. The pH was maintained at 6.0 by automatic feeding of a 2 M NaOH solution. The temperature, pH, agitation, and dissolved oxygen concentration were monitored and controlled using a CNS control system. A feeding solution comprising 600 g/L glucose and 40 g/L yeast extract was added at a feeding rate of < 5 g/L/h after the glucose was completely consumed.

### Metabolite extraction and analysis

To extract squalene, yeast cells were harvested by centrifugation at 13,000×*g* for 5 min to obtain an equivalent OD_600_ of 10. The harvested cells were resuspended in 600 µL of a 1:1 methanol–acetone (MA) solution with lysing matrix C. Subsequently, the mixture was mechanically disrupted using a FastPrep-24 5G homogenizer (MP Biomedicals, CA, USA) according to the manufacturer’s instructions. After filtration using 0.2-μm syringe filters, squalene extracted from the MA solution was analyzed using an Agilent high-performance liquid chromatography (HPLC) system equipped with a UV detector at 203 nm. Squalene was separated on a Kinetex 5 µm EVO C18 column (Phenomenex, Aschaffenburg, Germany) at 30 °C with an isocratic elution flow rate of 1.0 mL/min for 30 min. The mobile phase comprised acetonitrile, methanol, and water at a ratio of 90:9:1 (v/v).

Glucose, glycerol, ethanol and organic acid concentrations were determined with an HPLC system equipped with an Aminex HPX-87G column (Bio-Rad, CA, USA). In detail, a 1 mL culture sample was centrifuged and filtered through a 0.2-µm syringe filter and then analyzed on the HPLC system with 5 mM H_2_SO_4_ as the mobile phase at a flow rate of 0.6 mL/min at 45 °C for 30 min.

### Quantification of acetyl-CoA and TAG

Acetyl-CoA and TAG were quantified as described previously, with minor modifications [45–48]. Briefly, yeast cells were grown in YSC medium overnight at 30 °C with shaking at 250 rpm. The precultures were then inoculated into 50 mL of YSC medium containing 2% (w/v) glucose at an initial OD_600_ of 0.5 and cultivated for 6 days at 30 °C with shaking at 250 rpm. At appropriate timepoints, yeast cells were harvested by centrifugation at 13,000 rpm for 5 min at 4 °C.

For quantification of acetyl-CoA, 10 OD_600_ of cells were resuspended in 500 μL of a cold Tris-EDTA (pH 8.0) buffer solution (Sigma-Aldrich, Missouri, USA). Extracts were prepared with a FastPrep-24 5G homogenizer (MP Biomedicals, OH, USA) using lysing matrix C. After centrifuging at 13,000 rpm for 5 min at 4 °C, the supernatant was used for acetyl-CoA measurement with a PicoProbe™ Acetyl-CoA Fluorometric Assay Kit (Biovision, Milpitas, USA) according to the manufacturer's instructions. Acetyl-CoA concentrations were measured by fluorescence intensity at wavelengths 535 nm (excitation) and 587 nm (emission) on a microplate reader (SpectraMax Gemini XPS, Molecular Devices, CA, USA). For quantification of TAG, 2 OD_600_ of cells were homogenized in 1 mL of 5% NP-40 (Sigma-Aldrich, Missouri, USA). Using the homogenates, TAG was quantified using a Triglyceride Quantification Kit (BioVision, Milpitas, USA) according to the manufacturer's instructions.

### Confocal fluorescence microscopy

To analyze the size and number of LDs, yeast cells were grown in YSC medium supplemented with 2% (w/v) glucose. After growing for 24 h at 30 °C with shaking at 250 rpm, the cells were washed twice with phosphate-buffered saline (PBS) and fixed with 3.7% formaldehyde in PBS as described previously [11, 49, 50]. Next, the cells were stained with 5 µM BODIPY 493/503 dye for 30 min at 30 °C. Then, the cells were washed twice with PBS and observed with a Zeiss-LSM 780 multiphoton confocal microscope (Zeiss, AG, Germany) equipped with a Plan-Apochromat 63x/1.4 NA oil immersion objective. Confocal images were analyzed using ImageJ software (National Institutes of Health, Bethesda, USA) and ZEN imaging software (Zeiss, AG, Germany).

### Figure preparation

Figures were prepared using BioRender.Com for scientific illustrations.

## Supplementary Information

**Additional file 1: Figure S1.** Squalene production of engineered strains in which the *tHMG1* gene was integrated into a multicopy δ-sequence of the SQ00 genome. **Figure S2. **Differential interference contrast and confocal fluorescence microscopy images of the LDs in the LD metabolism-engineered strains. **Table S1**. List of plasmids and strains used in this study. **Table S2. **Sequence of N-degron tag. **Table S3. **Squalene production of the engineered strains in which Erg1 activity was partially inhibited by N-degron tag. **Table S4. **Squalene production of the LD metabolism-engineered strains.
