# Supplementary material for: Metabolic recycling of storage lipids promotes squalene biosynthesis in yeast
Source: Biotechnol Biofuels Bioprod. 2022 Oct 12;15:108. doi: 10.1186/s13068-022-02208-9 (PMC9555684; doi:10.1186/s13068-022-02208-9)
Supplement: Supplementary file 1 — Additional file 1: Figure S1. Squalene production of engineered strains in which the tHMG1 gene was integrated into a multicopy δ-sequence of the SQ00 genome. Figure S2. Differential interference contrast and confocal fluorescence microscopy images of the LDs in the LD metabolism-engineered strains. Table S1. List of plasmids and strains used in this study. Table S2. Sequence of N-degron tag. Table S3. Squalene production of the engineered strains in which Erg1 activity was partially inhibited by N-degron tag. Table S4. Squalene production of the LD metabolism-engineered strains. [file 13068_2022_2208_MOESM1_ESM.docx]

**Supplementary material for:**

***Metabolic recycling of storage lipids promotes squalene biosynthesis in yeast***

**Table of Contents**

**Supplementary Figure 1.** Squalene production of engineered strains in which the *tHMG1* gene was integrated into a multicopy δ-sequence of the SQ00 genome

**Supplementary Figure 2.** Differential interference contrast and confocal fluorescence microscopy images of the LDs in the LD metabolism-engineered strains.

## Supplementary Table 1. List of plasmids and strains used in this study.

**Supplementary Table 2.** Sequence of N-degron tag

**Supplementary Table 3.** Squalene production of the engineered strains in which Erg1 activity was partially inhibited by N-degron tag

**Supplementary Table 4.** Squalene production of the LD metabolism-engineered strains

**
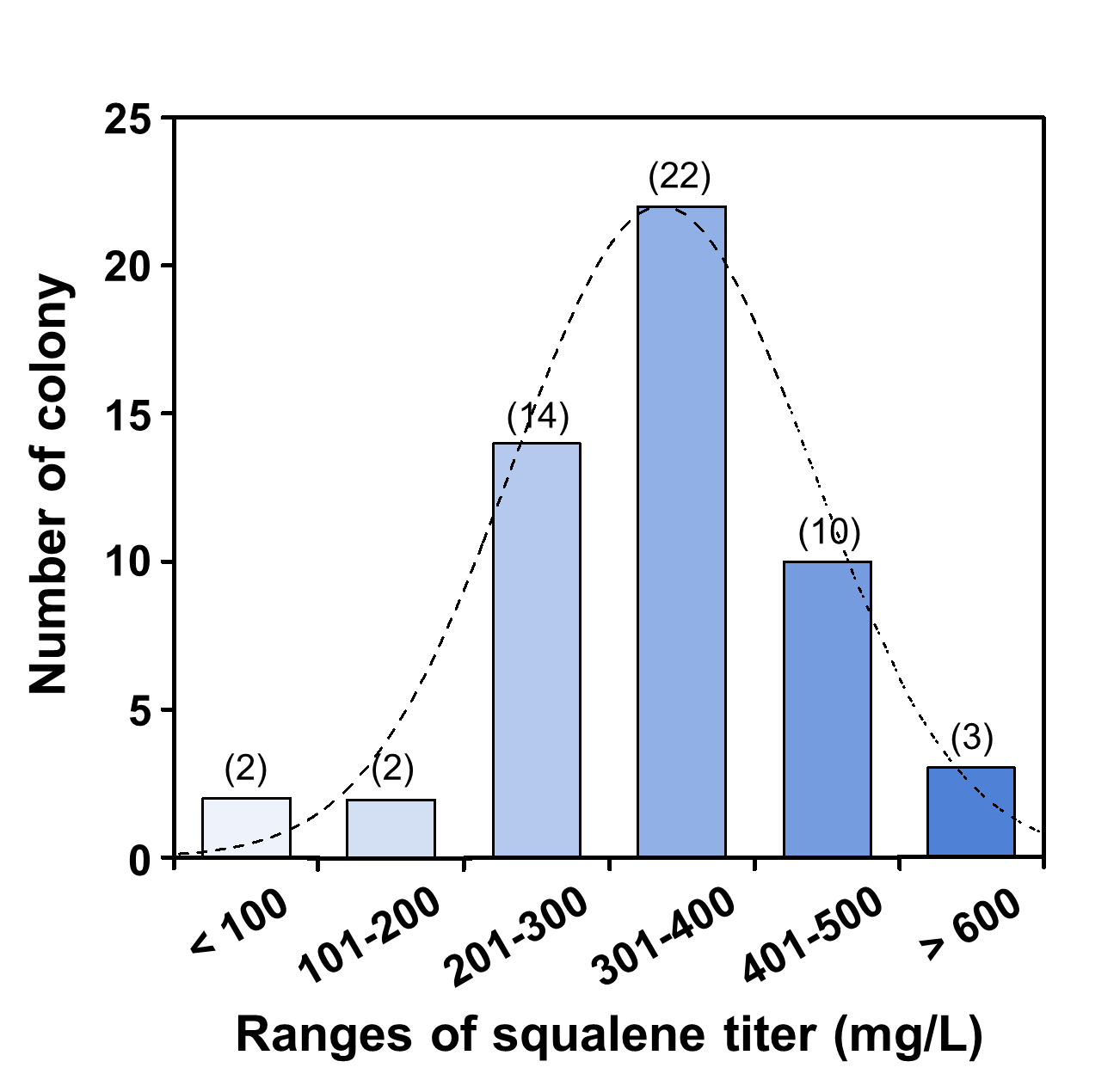
**

**Supplementary Figure 1. Squalene production of engineered strains in which the *tHMG1* gene was integrated into a multicopy δ-sequence of the SQ00 genome.** The numbers in parentheses indicate the colony number in respective range of the squalene titer. Yeast cells were grown in shake flasks with YSC minimal medium with 2% (w/v) glucose at 30 °C.


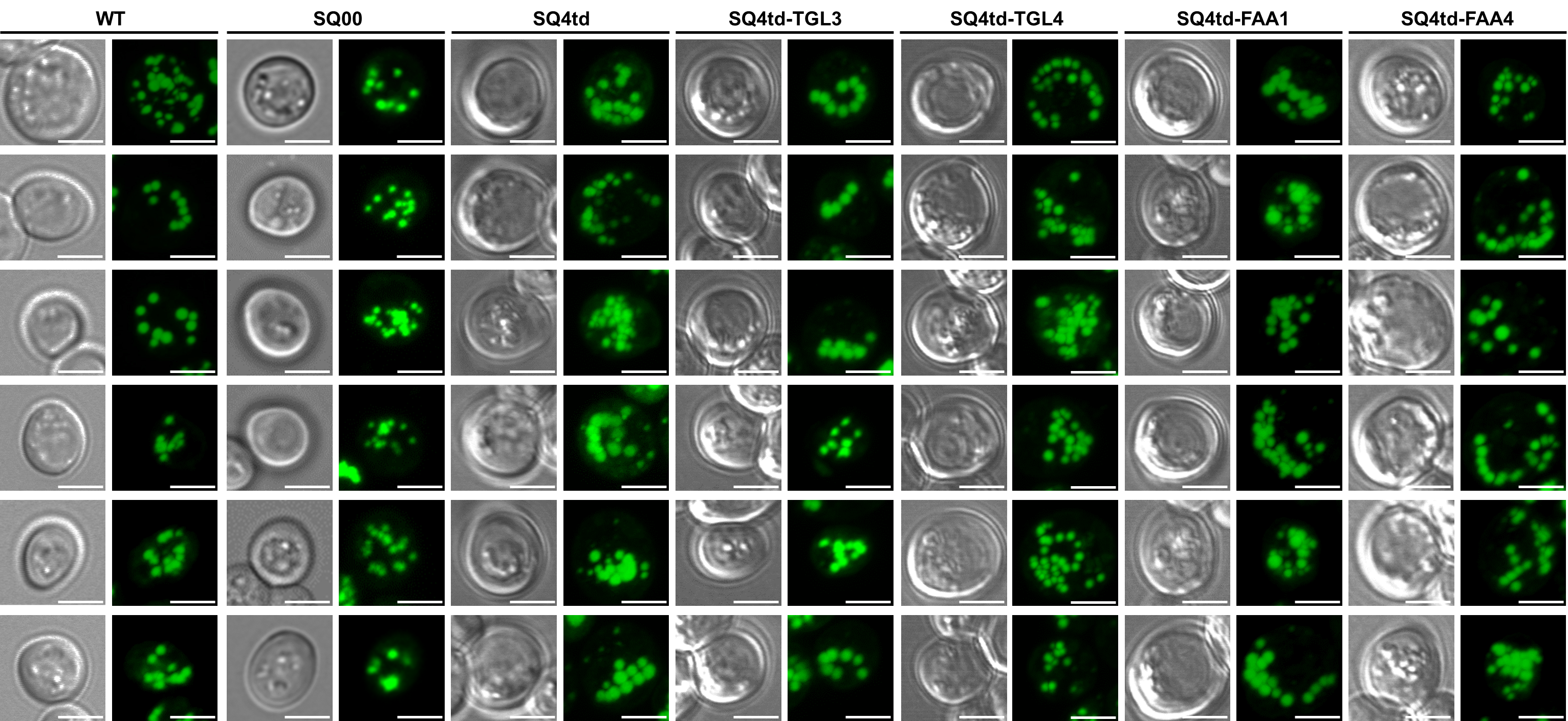


**Supplementary Figure 2.** **Differential interference contrast and confocal fluorescence microscopy images of the LDs in the LD metabolism-engineered strains.** The cells were grown in YSC medium with 2% (w/v) glucose at 30 °C, stained with BODIPY dye, and examined by confocal fluorescence microscopy. Scale bar, 2.5 µm.

## Supplementary Table 1. List of plasmids, strains, and primers used in this study

| **Plasmids** | | |
| --- | --- | --- |
| **Plasmids** | **Description / Genotype** | **Reference** |
| pUC57-URA3 | pUC57-based vector containing Amp^R^, *3 × Myc-URA3*, *CYC1t* | (Lee et al., 2015) |
| pUC57-GPD | pUC57-URA3 vector containing *GPD* promoter | (Lee et al., 2015) |
| pUC57-GPD-tHMG1 | pUC57-GPD harboring *tHMG1* (*P_GPD_*-*tHMG1*-*CYC1t*) | This study |
| pUC57-Erg1p-Degron | pUC57-URA3 vector harboring a replacement cassette for N-degron tag {*P_ERG1_-UBI4-Degron(K3K15)*} | This study |
| pUC57-CCW12 | pUC57-URA3Myc vector containing *CCW12* promoter | (Lee et al., 2015) |
| **Strains** | | |
| **Strains** | **Description / Genotype** | **Reference** |
| CEN.PK2-1D | *MATα ura3-52 trp1-289 leu2-3,112* *his3*Δ*1 MAL2-8^C^ SUC2* | Euroscarf |
| SQ00 | CEN.PK2-1D Δ*leu2::P_TEF1_-ERG20-T_CYC1_ P_INO2_::P_PGK1_* | (Kim et al., 2019) |
| SQ1t | SQ00 strain with random integration of *P_GPD_-tHMG1* at delta site (1 copy) | This study |
| SQ2t | SQ00 strain with random multiple integration of *P_GPD_-tHMG1* at delta sites (2 copies) | This study |
| SQ3t | SQ00 strain with random multiple integration of *P_GPD_-tHMG1* at delta sites (3 copies) | This study |
| SQ4t | SQ00 strain with random multiple integration of *P_GPD_-tHMG1* at delta sites (4 copies) | This study |
| SQ5t | SQ00 strain with random multiple integration of *P_GPD_-tHMG1* at delta sites (5 copies) | This study |
| SQ4td | SQ4t *P_ERG9_::P_ERG1_-UBI4-Degron(K3K15)* | This study |
| SQ4td-TGL3 | SQ4td *P_TGL3_::P_CCW12_* | This study |
| SQ4td-TGL4 | SQ4td *P_TGL4_::P_CCW12_* | This study |
| SQ4td-FAA1 | SQ4td *P_FAA1_::P_CCW12_* | This study |
| SQ4td-FAA4 | SQ4td *P_FAA4_::P_CCW12_* | This study |
| **Primers** | | |
| **Primer name** | **Primer sequence (5’-3’)** | |
| Del-delta_seq-  tHMG1-F | TGTTAGAAGATGACGCAAATGATGAGAAATAGTCATCTAAATTAGTGGAACCAGTCACGACGTTGTAAAA | |
| Del-delta_seq-  tHMG1-R | AAGGCTATAATATTAGGTATACAGAATATACTAGAAGTTCTCCTCGAGGAAGGTTTCCCGACTGGAAAGC | |
| Erg1p-to-Erg1-Ndeg-F | GCACCAGCAAAGAGGCGGTGCAATCATCAGTAATACAGGACTTGTCTCGCAAGATACGACAGGTTTCCCGACTGGAAAGC | |
| Erg1p-to-Erg1-Ndeg-R | TCGCATCGTAGGTAATTGTGTTGTCGGCATTAATCAATTCAGGTGCAACGTTAACAGCAGAACCAGAACCCTTAACCAAAGAAAC | |
| TAF10_qPCR-F | ATATTCCAGGATCAGGTCTTCCGTAGC | |
| TAF10_qPCR-R | GTAGTCTTCTCATTCTGTTGATGTTGTTGT | |
| tHMG1_qPCR-F | AGAACTTAGTTTCGACGGATTCT | |
| tHMG1_qPCR-R | GGTGACTTCAGTCTTCACCAA | |
| Tgl3_Pro_Rep_  CCW12-F | AATAGGGTATGTTCTTTATAGGTCATTTTTACTTTTTTAGTAGGGCATTTCAGGAAACAGCTATGACCATGATTAC | |
| Tgl3_Pro_Rep_  CCW12-R | AGGGTCGGTATTACAGCAGACACCTTGTATTCCTGCGCCGTTTCCTTCATTATTGATATAGTGTTTAAGCGAATGACAGAAGAT | |
| Tgl4_Pro_Rep_  CCW12-F | TATGAAACGCTAAACCACAATTATAAAGTATGATTGATTCAAATTGTCATTATGTCATTAAGGTTTCCCGACTGGAAAGC | |
| Tgl4_Pro_Rep_  CCW12-R | CAAGGAGGGGCTTATTTTGTGTAGATGTAAGATCTGATATTTTGCTGCTCATGGTGGCTATTGATATAGTGTTTAAGCGAATGAC | |
| Faa1_Pro_Rep_  CCW12-F | TGCGTTGTAGATGTAAGACCGATCCACTTTGCCAGCTGCTTACGCTGCGGAAAGTAGGTTTCCCGACTGGAAAGC | |
| Faa1_Pro_Rep_  CCW12-R | GTTTCATGCTCATTGGCGGCTTTCCCAACTGGAACGGTATATTGAGCAACCATTATTGATATAGTGTTTAAGCGAATGAC | |
| Faa4_Pro_Rep_  CCW12-F | TTATCCAACACGATGGAGCGTGTTGGGCGCTTAAAAAGAGACTCACTAACTGTTCAGGTTTCCCGACTGGAAAGC | |
| Faa4_Pro_Rep_ CCW12-R | GTTTCATGCTCATTGTCGGCTTCGCCAACTGCAACGGAATATTGTTCGGTCATTATTGATATAGTGTTTAAGCGAATGAC | |

**Supplementary Table 2**. Sequence of N-degron tag

| **Plasmid name** | **Sequence (5’-3’)** |
| --- | --- |
| pUC57-Erg1p-Degron  *P_ERG1_-*UBI4-Degron(K3K15) | TACGTTCGGGATTTAATCTTCTCGCAGTCTGCAGGCGCCTTGAGATTTGCGTTCGGCCTAAACGTTTGCTCCACAAACGTGAATGGTATGAACATGGACATGAGCGTGGTTCAGGGCACTCTACGGGATCGTGGCGAATGGGAATCGTTCTGCAAGCTCTTCTACCAAACCATCGGCGAATTTGCGTCGCTTTAATGCGATACTGCCGTAGCGGGCCTTCGTATAGCTCGGCCGAGCTCGTACAAAAGGCAAGCAGTGTATCGGACAGAGCTGATATAACACAATACGCTCGTAGTCGATGCATGCCGTGGCTGCTCTCGGTCGGGTATAAGTCTTAGACAATAGTCTTACCTCGCATGTATAATAAATCTTTTGTATTTAATCTATTATATGTTTCTATGCTTTTTTTTCCTATTGTTGTTTGCTTTTCCTTTTCCTTATTTCTTTCTAGCTTCTAATTTTCTTTCTTTTTTTTTTTTTTTTCATTGAAAATTATATATATATATATATATCAGAACAATTGTCCAGTATTGAACAATACAGGTTATTTCGAACAATTGAAAAAAAAAAATCACAGAAAAACATATCGAGAAAAGGGTCATGCAGATTTTCGTCAAGACTTTGACCGGTAAAACCATAACATTGGAAGTTGAATCTTCCGATACCATCGACAACGTTAAGTCGAAAATTCAAGACAAGGAAGGTATCCCTCCAGATCAACAAAGATTGATCTTTGCCGGTAAGCAGCTAGAAGACGGTAGAACGCTGTCTGATTACAACATTCAGAAGGAGTCCACCTTACATCTTGTGCTAAGGCTAAGAGGTGGTTTCCACAAATCTGGTGCTTGGTTGTTGCCAGTTTCTTTGGTTAAGGGTTCTGGT |

**Supplementary Table 3. Squalene production of the engineered strains in which Erg1 activity was partially inhibited by N-degron tag**

| **Strain** | **Time (h)** | **Without terbinafine** | | | | | | | |
| --- | --- | --- | --- | --- | --- | --- | --- | --- | --- |
|  |  | **Cell growth**  **(OD_600_)** | | | **Squalene titer**  **(mg/L)** | | **Squalene**  **contents**  **(mg/g DCW)** | | |
| **WT** | 72 | 20.3 (±0.54) | | | 2.01 (±0.64) | | 0.20 (±0.06) | | |
|  | 144 | 19.1 (±1.11) | | | 2.01 (±0.05) | | 0.21 (±0.02) | | |
| **SQ00** | 72 | 19.2 (±1.23) | | | 2.10 (±1.04) | | 0.23 (±0.11) | | |
|  | 144 | 19.2 (±0.56) | | | 4.14 (±0.53) | | 0.46 (±0.07) | | |
| **SQ4t** | 72 | 20.48 (±0.54) | | | 410.46 (±9.86) | | 40.10 (±1.52) | | |
|  | 144 | 21.29 (±0.85) | | | 550.89 (±6.71) | | 51.81 (±2.67) | | |
| **SQ4td** | 72 | 21.39 (±0.94) | | | 711.84 (±13.64) | | 66.85 (±9.73) | | |
|  | 144 | 20.31 (±0.67) | | | 720.47 (±22.81) | | 85.24 (±2.14) | | |
|  | | |  | | | | | | |
| **Strain** | **Time (h)** | **With terbinafine** | | | | | | | |
|  |  | **Concentration of terbinafine (µg/mL)** | | **Cell growth (OD_600_)** | | **Squalene titer (mg/L)** | | **Squalene contents**  **(mg/g DCW)** |  |
| **SQ4t** | 72 | 10 | | 19.61  (±0.60) | | 449.87  (±66.25) | | 46.04  (±8.31) |  |
|  | 144 |  |  | 20.36  (±1.07) | | 575.35  (±75.30) | | 56.53  (±6.90) |  |
|  | 72 | 30 | | 18.94  (±0.24) | | 409.10  (±58.58) | | 43.20  (±6.28) |  |
|  | 144 |  |  | 19.45  (±0.67) | | 511.75  (±34.14) | | 52.60  (±1.84) |  |
|  | 72 | 50 | | 18.06  (±0.77) | | 257.82  (±17.19) | | 28.61  (±2.66) |  |
|  | 144 |  |  | 18.55  (±0.49) | | 364.41  (±60.27) | | 39.21  (±5.55) |  |

**Supplementary Table 4. Squalene production of the LD-metabolism engineered strains**

| **Strain** | **Time**  **(h)** | **Cell growth**  **(OD_600_)** | **Squalene titer**  **(mg/L)** | | **Squalene contents**  **(mg/g DCW)** |
| --- | --- | --- | --- | --- | --- |
| **WT** | 24 | 16.49 (±0.27) | | 0.25 (±0.23) | 0.03 (±0.03) |
|  | 48 | 21.15 (±0.36) | | 2.15 (±0.01) | 0.20 (±0.00) |
|  | 72 | 20.30 (±0.54) | | 2.01 (±0.64) | 0.20 (±0.06) |
|  | 144 | 19.11 (±1.11) | | 2.01 (±0.05) | 0.21 (±0.02) |
| **SQ00** | 24 | 17.68 (±1.47) | | 0.28 (±0.00) | 0.03 (±0.03) |
|  | 48 | 15.22 (±1.56) | | 0.78 (±0.18) | 0.11 (±0.04) |
|  | 72 | 19.23 (±1.23) | | 1.83 (±0.37) | 0.22 (±0.11) |
|  | 144 | 19.19 (±0.56) | | 2.76 (±0.84) | 0.43 (±0.06) |
| **SQ4td** | 24 | 16.38 (±0.46) | | 322.23 (±34.86) | 39.40 (±4.83) |
|  | 48 | 19.04 (±0.15) | | 601.14 (±39.43) | 63.14 (±3.64) |
|  | 72 | 21.39 (±0.94) | | 711.84 (±13.64) | 66.85 (±9.73) |
|  | 144 | 20.31 (±0.67) | | 720.47 (±22.81) | 85.24 (±2.14) |
| **SQ4td-TGL3** | 24 | 14.44 (±1.41) | | 340.09 (±52.48) | 47.20 (±7.07) |
|  | 48 | 18.57 (±0.30) | | 738.60 (±17.23) | 79.55 (±2.22) |
|  | 72 | 21.66 (±0.73) | | 832.12 (±51.67) | 76.86 (±4.29) |
|  | 144 | 21.26 (±1.16) | | 862.62 (±48.26) | 117.38 (±8.99) |
| **SQ4td-TGL4** | 24 | 18.01 (±0.83) | | 331.57 (±57.68) | 36.67 (±4.85) |
|  | 48 | 19.18 (±2.71) | | 729.66 (±20.97) | 77.01 (±10.26) |
|  | 72 | 21.57 (±1.22) | | 801.95 (±38.06) | 74.61 (±7.14) |
|  | 144 | 15.31 (±1.17) | | 816.48 (±66.32) | 106.70 (±5.68) |
| **SQ4td-FAA1** | 24 | 17.06 (±0.94) | | 337.37 (±34.32) | 39.71 (±5.53) |
|  | 48 | 19.08 (±0.22) | | 658.04 (±19.73) | 68.99 (±2.45) |
|  | 72 | 20.51 (±0.48) | | 706.52 (±17.92) | 68.88 (±0.71) |
|  | 144 | 17.41 (±0.62) | | 774.07 (±21.43) | 88.98 (±4.21) |
| **SQ4td-FAA4** | 24 | 17.73 (±0.97) | | 328.17 (±30.79) | 37.20 (±5.48) |
|  | 48 | 20.83 (±0.95) | | 601.72 (±48.41) | 57.87 (±5.50) |
|  | 72 | 22.14 (±0.73) | | 664.54 (±31.62) | 60.13 (±4.83) |
|  | 144 | 15.94 (±0.73) | | 723.90 (±32.73) | 90.99 (±5.99) |
| **SQ4td-TGL3/4** | 24 | 17.21 (±0.70) | | 322.73 (±16.27) | 37.52 (±1.88) |
|  | 48 | 20.83 (±1.66) | | 662.62 (±12.79) | 63.85 (±4.03) |
|  | 72 | 20.13 (±1.49) | | 810.73 (±24.69) | 80.93 (±8.08) |
|  | 144 | 19.21 (±1.62) | | 832.63 (±48.68) | 87.02 (±7.62) |

**References**

Kim, J. E., Jang, I. S., Son, S. H., Ko, Y. J., Cho, B. K., Kim, S. C., & Lee, J. Y. (2019). Tailoring the *Saccharomyces cerevisiae* endoplasmic reticulum for functional assembly of terpene synthesis pathway. *Metab Eng*, *56*, 50-59.

Lee, J. Y., Kang, C. D., Lee, S. H., Park, Y. K., & Cho, K. M. (2015). Engineering cellular redox balance in *Saccharomyces cerevisiae* for improved production of L-lactic acid. *Biotechnol Bioeng*, *112*(4), 751-758.
